# Deep Sequencing Uncovers Caste-Associated Diversity of Symbionts in the Social Ant Camponotus japonicus

**DOI:** 10.1128/mBio.00408-20

**Published:** 2020-04-21

**Authors:** Akiko Koto, Masaru Konishi Nobu, Ryo Miyazaki

**Affiliations:** a Bioproduction Research Institute, National Institute of Advanced Industrial Science and Technology (AIST), Tsukuba, Japan; b Computational Bio Big Data Open Innovation Laboratory (CBBD-OIL), AIST, Tokyo, Japan; c Faculty of Life and Environmental Sciences, University of Tsukuba, Tsukuba, Japan; Corporación CorpoGen

**Keywords:** ant, gut microbiota, social insect

## Abstract

Symbiotic microorganisms can have a profound impact on the host physiology and behavior, and novel relationships between symbionts and their hosts are continually discovered. A colony of social ants consists of various castes that exhibit distinct lifestyles and is, thus, a unique model for investigating how symbionts may be involved in host eusociality. Yet our knowledge of social ant-symbiont dynamics has remained rudimentary. Through 16S rRNA gene deep sequencing of the carpenter ant Camponotus japonicus symbiont community across various castes, we here report caste-dependent diversity of commensal gut microbiota and lineage divergence of “*Candidatus* Blochmannia,” an obligate endosymbiont. While most prevalent gut-associated bacterial populations are found across all castes (*Alphaproteobacteria*, *Gammaproteobacteria*, *Bacteroidetes*, and *Cyanobacteria*), we also discovered uncultured populations that are found only in males (belonging to *Corynebacteriales*, *Alkanindiges*, and *Burkholderia*). Most of those populations are not detected in laboratory-maintained queens and workers, suggesting that they are facultative gut symbionts introduced via environmental acquisition. Further inspection of “*Ca.* Blochmannia” endosymbionts reveals that two populations are dominant in all individuals across all castes but that males preferentially contain two different sublineages that are diversified from others. Clearly, each caste has distinct symbiont communities, suggesting an overlooked biological aspect of host-symbiont interaction in social insects.

## Abstract (precis)

Social animals, such as primates and some insects, have been shown to exchange symbiotic microbes among individuals through sharing diet or habitats, resulting in increased consistency of microbiota among social partners. The ant is a representative of social insects exhibiting various castes within a colony; queens, males, and nonreproductive females (so-called workers) show distinct morphologies, physiologies, and behaviors but tightly interact with each other in the nest. However, how this social context affects their gut microbiota has remained unclear. In this study, we deeply sequenced the gut symbiont community across various castes of the carpenter ant Camponotus japonicus. We report caste-dependent diversity of commensal gut microbial community and lineage divergence of the mutualistic endosymbiont “*Candidatus* Blochmannia.” This report sheds light on the hidden diversity in microbial populations and community structure associated with guts of males in social ants.

## OBSERVATION

The importance of symbiont diversity is becoming increasingly recognized as a key component of the host’s physiology and behavior (1, 2). For primates and social insects, symbiotic microbes have been shown to be exchanged among individuals in social interactions (e.g., through sharing diet or habitats), resulting in increased consistency between microbiota among social partners (3–5). However, disentangling how those social contexts affect the diversity of gut microbiota remains challenging.

Ants are eusocial insects that can serve as model organisms for social interactions given their clear social hierarchy. An ant colony is, in principle, composed of reproductive castes (queens and males) and a nonreproductive caste (workers), each of which shows different morphologies, physiologies, and behaviors (6). For carpenter ants of the *Camponotus* species, newly emerged queens and males mate in the nuptial flight. The inseminated queens construct a nest to lay eggs, and most of the newly hatched ants are raised as workers, consisting of a nonreproductive female caste showing task allocations distributed in an age-dependent and environment-dependent manner (6, 7). While workers forage outside and then feed nest mates, queens and males remain inside the nest until the nuptial flight. These castes also display differences in longevity—queens can live for over 20 years (6, 8), workers live for between a month and a few years, and males die immediately after the mating flight. Given the diversity among castes, *Camponotus* species can be a suitable model to explore how social contexts, particularly castes and colonies, affect gut microbiota.

The forms of diversity in the commensal gut microbiota remain unclear due to the high abundance of endosymbiotic “*Candidatus* Blochmannia” in gut-associated cells (9–11) overwhelming those of commensal gut symbionts in DNA-based analyses (e.g., more than 95% of the reads of *Camponotus* gut metagenome were assigned to “*Ca.* Blochmannia” [12, 13]). While “*Ca.* Blochmannia” undergoes maternal transmission to offspring and interacts mutualistically with the host (14), whether host-endosymbiont interactions differ between castes also remains unknown.

**FIG 1:**
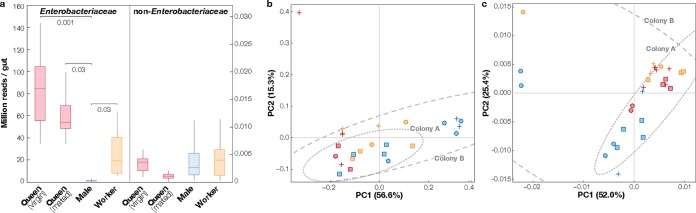
Differences in gut community composition between C. japonicus carpenter ants of various castes and colonies in the field. (a) Box plot of the estimated abundances of *Enterobacteriaceae* (left) and non-*Enterobacteriaceae* (right) symbionts per gut. The abundance of *Enterobacteriaceae* symbionts was estimated by qPCR using specific primers for “*Ca.* Blochmannia.” The abundance of non-*Enterobacteriaceae* symbionts was calculated with the estimated abundance of *Enterobacteriaceae* multiplied by the relative abundance of non-*Enterobacteriaceae* reads in amplicon sequencing. *P* values are indicated. (b) Principal-coordinate analysis for the weighted UniFrac distance of non-*Enterobacteriaceae* microbial community. Samples with at least 57 reads belonging to non-*Enterobacteriaceae* clades were subsampled and plotted. Samples with at least 788 reads belonging to minor *Enterobacteriaceae* clades were subsampled and plotted. For each sample, the colony (colony A = square, colony B = circle, others = plus sign) and caste (virgin and mated queens = red, workers = orange, males = blue) are indicated. 99% confidence ellipses are shown for colony A and colony B. (c) Data representing minor *Enterobacteriaceae* clades (excluding two dominant OTUs) are shown as described for panel b.

Here, we explored and compared the gut microbial communities of various castes (virgin or mated queen, worker, and male) of the carpenter ant C. japonicus through deep sequencing of the bacterial 16S rRNA gene. We collected a total of 39 individual ants in Tsukuba, Japan, in the following categories: 7 virgin queens, 4 mated queens, 7 workers, and 10 males from eight field colonies and 2 mated queens and 9 workers from two laboratory-reared colonies (see Text S1 in the supplemental material). The amplicon sequencing of individual gut samples yielded 101,961,345 sequences (average, 2.6 × 10^6^ sequences per sample) associated with 756 operational taxonomic units (OTUs). A major portion of the sequences (100,843,490) were affiliated with endosymbiotic *Enterobacteriaceae*, most (99.999%) of which primarily consisted of 2 dominant “*Ca.* Blochmannia” populations (46.1 million reads for CJS630 and 54.1 million reads for CJS647) and 261 other minor “*Ca.* Blochmannia” populations (0.6 million reads). Using results of quantitative PCR (qPCR) analyses of 16S rRNA gene copies (see Fig. S1 in the supplemental material), we found that the estimated abundance of “*Ca.* Blochmannia” was much lower in males than in other castes (*P < *0.05; only field samples compared), while the abundances of commensal gut microbiota (i.e., non-*Enterobacteriaceae*) were statistically indistinguishable among castes (*P > *0.1) (Fig. 1A).

10.1128/mBio.00408-20.1 TEXT S1 Supplemental methods. Download Text S1, DOCX file, 0.03 MB. Copyright © 2020 Koto et al. 2020 Koto et al. This content is distributed under the terms of the Creative Commons Attribution 4.0 International license.

10.1128/mBio.00408-20.2 FIG S1 Quantification of 16S rRNA gene copies of “*Ca.* Blochmannia” in each individual C. japonicus sample. Absolute abundances of 16S rRNA gene copies in individual guts were assessed by qPCR using “*Ca.* Blochmannia”-specific primers. Individual samples were obtained from the field or from laboratory-maintained colonies. Each individual’s caste and sample identifier (ID) are indicated. The colony ID is shown at the bottom of each bar. Queen, worker, and male are depicted in red, green, and blue bars, respectively. Download FIG S1, TIF file, 1.5 MB. Copyright © 2020 Koto et al. 2020 Koto et al. This content is distributed under the terms of the Creative Commons Attribution 4.0 International license.

**FIG 2:**
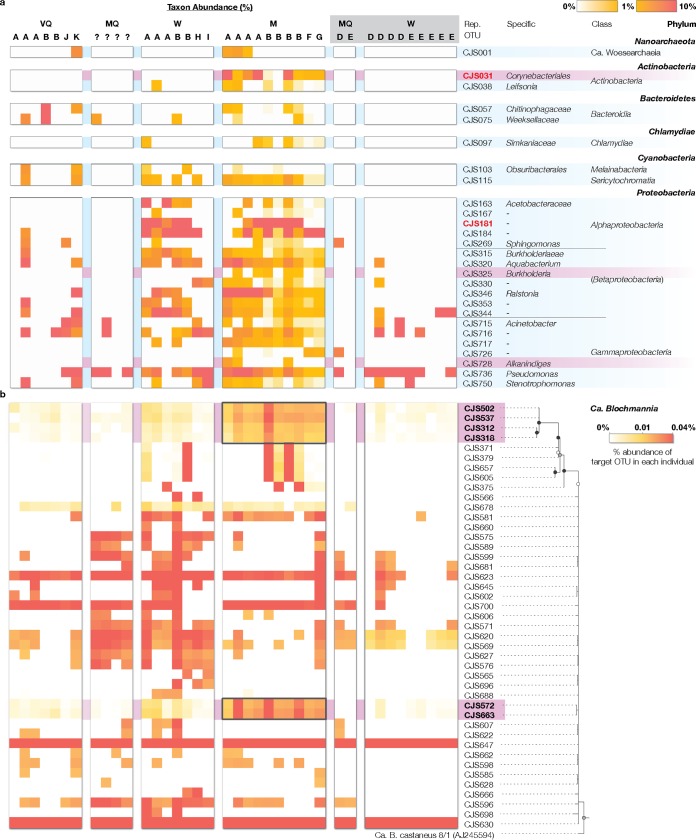
Phylogeny, distribution, and relative abundance of symbionts across various castes of C. japonicus. (a) Relative abundance of commensal gut microbes. OTUs were grouped based on 97% sequence similarity (CD-HIT-EST v4.8.1). The relative abundance of each OTU group in non-*Enterobacteriaceae* in each individual is shown along with each individual’s caste (VQ = virgin queen, MQ = mated queen, W = worker, M = male) and colony source (colonies A to K, with unknown colonies indicated as “?”). Individuals belonging to colonies maintained in the laboratory are highlighted in gray. Only OTU groups with at least 1% abundance in at least two individuals are included. For each OTU group, a representative OTU, phylum, class, and the most specific classification beyond class (genus, family, or order; if defined) are shown. OTU groups with the same classification are indicated with hyphens. OTUs associated only with males are highlighted in purple. OTUs are depicted in red letters if (i) the OTUs were detected across at least 70% of the individuals in a specific caste and (ii) the average abundance across that caste greater was than 5.6%—the lowest detection limit among queens—based on the data from the queen with the lowest nonzero number of reads for non-*Enterobacteriaceae* symbionts (18 reads for virgin queen G1; leftmost in figure). (b) Abundance and phylogenetic tree of “*Ca.* Blochmannia.” Only OTUs with at least 0.01% abundance in two or more individuals are shown. Sequences were aligned using SINA and SILVA v132. The phylogenetic tree was constructed using RAxML v8 with generalized time reversal (GTR) model, 4 discrete GAMMA categories, and 100 bootstrap iterations (see complete tree in Fig. S5). Bootstrap values are shown as colored circles on each branch (black = ≥90, gray = ≥75, and white = ≥50; none are shown for those with values of <50). Note that the OTUs are not intracellular variations of 16S rRNA gene sequences because “*Ca.* Blochmannia” has a single copy of a 16S rRNA gene. The abundance of each OTU in each individual is shown as described for panel a. Those found in statistically significantly higher abundance in males than in other castes (*P < *0.05) are marked in purple.

Our deep sequencing allowed detection of 1,117,855 sequences affiliated with commensal gut microbes spanning 421 OTUs (Fig. S2). We performed beta-diversity analysis of the commensal microbial communities to examine differences in their structures among castes (Text S1). The community structures indeed differed within colonies, whereas no obvious differences were observed between colonies (Fig. 1B; see also Fig. S3). The relative contribution of caste to the community structure was obvious: while queens tend to have consistent communities, workers and males had distinctive communities that were highly variable among individuals (Fig. 1B). All castes in the field had gut microbes belonging to *Gammaproteobacteria*, *Alphaproteobacteria*, *Cyanobacteria*, and *Bacteroidetes* (Fig. 2A), most of which were not detected in laboratory-reared samples. *Acetobacteraceae* populations (CJS181 and CJS184) frequently detected in field samples (12) were also undetected, indicating that most commensal microbial populations are facultative symbionts acquired from the environment. Several *Gammaproteobacteria* species were still visible in laboratory-born workers, suggesting the potential of vertical inheritance. Interestingly, while no clades were exclusive to queens or workers, three taxa were associated only with males (*Alkanindiges*, *Burkholderia*, and *Corynebacteriales*), suggesting that the male gut environment or behavior may harbor unique features that select for such organisms. As of yet, detailed studies on the behavior of males in the nest are still lacking. Currently conceivable routes of exposure to exogenous microbes include nest soil or trophallaxis with nest mates (6), but both are available to other castes. The male-specific microbes might thus be provided by further unknown mechanisms, such as specific diet or infection for males. The uncultured *Corynebacteriales* population (CJS031) was found to be present across nearly all male samples (9 of 10) and belongs to an uncharacterized lineage containing moderately related (≤96.2% sequence similarity) symbionts from Australian weaver ants (Polyrhachis robsoni) (Fig. S4) (15). The *Corynebacteriales* order contains a variety of environmental bacteria often degrading hydrocarbons (16) and secreting various metabolites (17, 18). Given the characteristics of these bacteria, we suspect that the male-specific microbes may be involved in the production of cuticular hydrocarbons, profiles of which often act as sensory cues to distinguish species, sexes, or social status (19–21).

10.1128/mBio.00408-20.3 FIG S2 Alpha rarefaction curve for non-*Enterobacteriaceae* reads. Observed OTU curves (generated using QIIME2 “diversity alpha-rarefaction” function) are shown for (a) field samples with greater than or equal to 57 non-*Enterobacteriaceae* reads (–p-max-depth 57) and (b) male and worker (field) samples with greater than or equal to 181 non-*Enterobacteriaceae* reads (–p-max-depth 181). Curves for each caste are indicated by color (red = queen, orange = worker, and blue = male). Download FIG S2, TIF file, 0.3 MB. Copyright © 2020 Koto et al. 2020 Koto et al. This content is distributed under the terms of the Creative Commons Attribution 4.0 International license.

10.1128/mBio.00408-20.4 FIG S3 Unweighted pair group method with arithmetic mean (UPGMA) sample clustering based on beta diversity. The diversities were calculated from (a) commensal gut symbiont microbial communities of field samples with at least 57 reads and (b) minor “*Ca.* Blochmannia” communities of field samples with at least 1,273 reads (i.e., excluding the two most dominant populations). Sample ID is indicated by color based on the caste (red = queen, blue = male, orange = worker). Colony ID is also indicated with a scale using different shades of gray. Support values are also shown (black circle, ≥90%; gray, ≥70%; white, ≥50%). The calculation was performed using QIIME2 (qiime diversity beta-rarefaction) and weighted normalized UniFrac (–p-metric weighted_normalized_unifrac) and UPGMA for clustering (–p-clustering-method upgma). Download FIG S3, TIF file, 0.3 MB. Copyright © 2020 Koto et al. 2020 Koto et al. This content is distributed under the terms of the Creative Commons Attribution 4.0 International license.

10.1128/mBio.00408-20.5 FIG S4 Phylogeny, distribution, and abundance of uncultured *Corynebacteriales* across various castes of C. japonicus. (Left) Neighbor-joining tree constructed using ARB with 1,000 bootstrap iterations. Sequences were aligned using SINA and SILVA v132. (Middle) The fraction of individuals of a specific caste and colony that harbored CJS031 with at least 0.01% abundance is shown. (Right) The average abundances of CJS031 across individuals from specific castes and colonies are shown. Castes of laboratory-maintained colonies are highlighted in gray. Download FIG S4, TIF file, 0.3 MB. Copyright © 2020 Koto et al. 2020 Koto et al. This content is distributed under the terms of the Creative Commons Attribution 4.0 International license.

10.1128/mBio.00408-20.6 FIG S5 Phylogeny, distribution, and abundance of “*Ca.* Blochmannia” across various castes of C. japonicus. Multiple outliers are included. Sequence alignment of the 16S rRNA gene used for drawing the tree is also shown. Sequences of populations that are male associated and ubiquitous across all field samples are highlighted (white and red, respectively). See Fig. 2 for more details. Download FIG S5, PDF file, 1.9 MB. Copyright © 2020 Koto et al. 2020 Koto et al. This content is distributed under the terms of the Creative Commons Attribution 4.0 International license.

“*Ca.* Blochmannia” populations were diverse in individual ants (Fig. 2B; see also Fig. S5), which is in agreement with the general aspects of endosymbiont genome evolution (22, 23). We found that CJS647 (53.43% of total microbial community on average), CJS630 (45.58%), and CJS623 (0.04%) were ubiquitous across castes and colonies in the field. Given that all “*Ca.* Blochmannia” genomes sequenced had a single copy of a 16S rRNA gene (24), each detected OTU likely represents distinct “*Ca.* Blochmannia” populations and not variation in 16S rRNA sequences in a single genome. This is further supported by unequal abundances of two dominant populations (CJS647 and CJS630) (Fig. S6). The two dominant populations were present even in all laboratory-reared samples, suggesting that they are fundamentally important for their symbiotic relationships with the host. While the presence of two different genotypes of “*Ca.* Blochmannia” has been previously reported at the colony level of polygyne species where the two genotypes are derived from two queens (25), this is the first report that two dominant “*Ca.* Blochmannia” phylotypes transmitted by a single queen cocolonized individual ants in monogyne colonies. More importantly, the community structures of the minor *Enterobacteriaceae* symbionts (excluding the two dominant populations CJS630 and CJS647) were found to differ between the male caste and other castes (Fig. 1C). While most “*Ca.* Blochmannia” populations were sporadically present across different castes and colonies, two specific sublineages containing CJS502 or CJS572 were found in all males at a higher relative abundance than in other castes (*P < *0.05), indicating that those lineages are associated with males rather than representing random inheritance due to the small inoculum size in males. In addition, one of those containing CJS502 showed a relatively high level of phylogenetic deviation from the others. The higher abundances of these populations in males than in other castes (7.1× to 12.4× and 4.4× to 6.3× higher than queens and workers, respectively) are unlikely to have a significant impact on the host given their relatively low abundance compared to the two dominant CJS647 and CJS630 populations. However, the abundance of these male-associated “*Ca.* Blochmannia” endosymbionts can collectively reach up to 151% higher than that of the remaining non-*Enterobacteriaceae* commensal gut symbionts. The pervasiveness and pronounced abundance of the minor “*Ca.* Blochmannia” populations suggest that they may be enriched through host development and/or may increase their fitness more in males than females to play potential roles in male-specific functions.

10.1128/mBio.00408-20.7 FIG S6 Abundances of two dominant “*Ca.* Blochmannia” populations in C. japonicus. The box plot shows the relative (percent) abundances of CJS647 and CJS630 in individual guts (*n* = 39). Note that their abundances are unequal (*P = *6.7 × 10^−39^). Download FIG S6, TIF file, 0.1 MB. Copyright © 2020 Koto et al. 2020 Koto et al. This content is distributed under the terms of the Creative Commons Attribution 4.0 International license.

Males in social insect seldom show social tasks and thus have not been the focus of analyses of social behavior or physiology. However, considering their central role in reproduction, one can expect that males have unique systems or life cycles to maximize their success in the nuptial flight. Our report provides the first glimpse into the uniqueness of microbial populations and community structure associated with male guts in social insects.

### Data availability.

The 16S rRNA gene amplicon data sets generated during this study have been deposited and are available in the Sequence Read Archives of the National Center for Biotechnology Information (NCBI), European Bioinformatics Institute (EBI), and DNA Data Bank of Japan (DDBJ) under accession no. SRR10569543 to SRR10569587.
