# Supplementary material for: Deep Sequencing Uncovers Caste-Associated Diversity of Symbionts in the Social Ant Camponotus japonicus
Source: mBio. 2020 Apr 21;11(2):e00408-20. doi: 10.1128/mBio.00408-20 (PMC7175090; doi:10.1128/mBio.00408-20)
Supplement: TEXT S1 [file mBio.00408-20-s0001.docx]

**Supplementary Methods**

**Ant samples**

Virgin queens, males, and workers of *C. japonicus* were collected in the entrance of eight different colonies at the day of nuptial flight in May 2017 in Ibaraki, Japan (N36°03’48.1” and E140°07’54.1”). The colonies were at least 100 m away from each other, which makes sure that they are different colonies. From each colony, 1-10 ants with different castes were randomly sampled. Wingless mated queens were collected on the ground just after the nuptial flight. Some of the mated queens were reared in the laboratory in the insect breeding dish (φ100×H40mm, SPL Life Sciences) with the mixture of honey and chicken egg solidified with agar. Queens and workers from two of the colonies reared in the laboratory were sampled in August 2017. A total of 39 individual ants (seven virgin queens, four mated queens, seven workers, and 10 males from the field, and two mated queens and nine workers from the laboratory) were used for the gut community analysis.

**Genomic DNA extraction**

The digestive tract from the crop to rectum was dissected from each individual ant using tweezers that had been sterilized with 70% ethanol at a day after the collection. Genomic DNA was extracted from each digestive tract using the manufacture’s procedure with FastDNA SPIN kit for soil (MP-Biomedicals). The quantity and quality of extracted DNA was measured with nanodrop2000 and gel electrophoresis.

**16S rRNA gene amplification and sequencing**

The V4 region of the bacterial 16S rRNA gene was amplified using a 10 ng of DNA template and primers 515F (5’-GTGCCAGCMGCCGCGGTAA-3’) and 806R (5’-GGACTACHVGGGTWTCTAAT-3’). PCR reactions were performed in 60 µl with Amplitaq Gold DNA polymerase (AmpliTaq Gold™ DNA Polymerase, LD (Low DNA) with Gold Buffer and MgCl_2_, Applied Biosystems) at an annealing temperature of 50°C for 32 cycles. The cycle number where amplifications were not saturated was determined by semi-quantitative PCR. The PCR products were purified using AMPure XP beads (Beckman Coulter) and eluted with 40 μl of distilled water. A 30 ng of the 1^st^ PCR product was used for subsequent short PCR with Illumina barcoded primers for 12 cycles. The 2^nd^ PCR products were purified with AMPure XP beads and eluted with 40 μl of distilled water. The concentration of PCR products was measured using nanodrop2000, and equal quantities of three samples were loaded in a lane (average 530 ng per lane) and sequenced by Illumina MiSeq 2x250 bp pair-end platform. Average 3.4 million reads were obtained per sample.

**16S rRNA-based community analysis**

The community structure of *C. japonicus* gut microbial communities were analyzed based on the 16S rRNA amplicon sequencing. Sequences were trimmed, assembled, denoised, and clustered into representative sequences used QIIME2 v2019.4 (qiime dada2 denoise-paired --p-trim-left-f 13 --p-trim-left-r 13 --p-trunc-len-f 160 --p-trunc-len-r 160 --p-trunc-q 15) [1]. Alpha rarefaction curves were calculated qiime diversity alpha-rarefaction (qiime diversity alpha-rarefaction). For beta diversity analysis (qiime diversity core-metric-phylogenetic), intersequence phylogenetic distance was considered by aligning representative sequences using mafft (qiime alignment mafft) and constructing a phylogenetic tree for the aligned sequences through FastTree (qiime phylogeny fasttree; qiime phylogeny midpoint-root). Phylogeny was determined for each representative sequence by extracting the region corresponding to the primers used for amplification from sequences in the Silva v132 database of sequences clustered based on 99% similarity (qiime feature-classifier extract-reads --p-f-primer GTGCCAGCMGCCGCGGTAA --p-r-primer GGACTACHVGGGTWTCTAAT) [2, 3], training a Naive Bayes classifier using these sequences and their assigned taxonomy in the Silva v132 database (qiime feature-classifier fit-classifier-naive-bayes), and classification via scikit-learn (qiime feature-classifier classify-sklearn). R v3.5.3 (https://www.r-project.org) was used to plot confidence ellipses in principal coordinate analyses (ggplot2) and calculate p-values (Welch’s t-test). Where relevant, OTUs were clustered based on 97% similarity using CD-HIT-EST v4.8.1 [4, 5].

**Phylogenetic analysis**

For maximum-likelihood tree construction, representative sequences for target OTUs were aligned against the SILVA v132 16S rRNA alignment using SINA v1.2.11 [6], relevant 16S rRNA sequences (≥1200 bp) from the SILVA v132 database were further selected, and a maximum-likelihood tree was calculated using RAxML v8 with the generalized time reversal (GTR) model, 4 discrete GAMMA categories, and 100 bootstrap iterations [7]. For neighbor-joining tree construction, a base tree was constructed using long 16S rRNA sequences (≥1200 bp) and aligned OTU sequences were added to the tree by parsimony in ARB [8]. Sequences with single-base differences were not considered to be sequencing or PCR errors. Illumina sequencers have error rates ≤0.1% (i.e., single-base errors would only be anticipated error if the amplified sequence length was four times longer) [9]. Moreover, sequencing errors are minimized through denoising. Sequences with single-base differences were consistently detected across multiple samples (*i.e.*, distinct PCR runs), suggesting they were not generated from PCR errors.

**Quantitative PCR (qPCR) analysis**

*Ca*. Blochmannia 16S rRNA gene copies were quantified with qPCR for the samples used in the amplicon sequencing, with specific primers previously designed [10]. The 150-bp PCR fragment amplified with 16SFor (5'-AGAATTCCAGGTGTAGCGGTG-3') and 16SRev (5'-TACGGCATGGACTACCAGGG-3') primers were cloned into pTA2 vector (TOYOBO), and used as the standards. Absolute number of 16S rRNA gene copy per gut was estimated using the total amount of DNA extracted from each sample.

**Data availability**

The 16S rRNA gene amplicon datasets generated during this study are deposited and available in the Sequence Read Archives of National Center for Biotechnology Information (NCBI), European Bioinformatics Institute (EBI) and DNA Data Bank of Japan (DDBJ), http://trace.ddbj.nig.ac.jp/DRASearch, under SRR10569543-SRR10569587.

**References (not listed in the main text)**

1. Bolyen E, Rideout JR, Dillon MR, Bokulich NA, Abnet CC, Al-Ghalith GA, et al. Reproducible, interactive, scalable and extensible microbiome data science using QIIME 2. Nature Biotechnology. 2019;37(8):852-857.

2. Quast C, Pruesse E, Yilmaz P, Gerken J, Schweer T, Yarza P, et al. The SILVA ribosomal RNA gene database project: improved data processing and web-based tools. Nucleic Acids Research. 2012;41(D1):D590-D596.

3. Yilmaz P, Parfrey LW, Yarza P, Gerken J, Pruesse E, Quast C, et al. The SILVA and “All-species Living Tree Project (LTP)” taxonomic frameworks. Nucleic Acids Research. 2014;42(D1):D643-D648.

4. Fu L, Niu B, Zhu Z, Wu S, Li W. CD-HIT: accelerated for clustering the next-generation sequencing data. Bioinformatics. 2012;28(23):3150-3152.

5. Li W, Godzik A. Cd-hit: a fast program for clustering and comparing large sets of protein or nucleotide sequences. Bioinformatics. 2006;22(13):1658-1659.

6. Pruesse E, Peplies J, Glöckner FO. SINA: Accurate high-throughput multiple sequence alignment of ribosomal RNA genes. Bioinformatics. 2012;28(14):1823-1829.

7. Stamatakis A. RAxML version 8: a tool for phylogenetic analysis and post-analysis of large phylogenies. Bioinformatics. 2014;30(9):1312-1313.

8. Westram R, Bader K, Prüsse E, Kumar Y, Meier H, Glöckner FO, et al. ARB: A Software Environment for Sequence Data. Handbook of Molecular Microbial Ecology I;10.1002/9781118010518.ch462011. p. 399-406.

9. Glenn TC. Field guide to next-generation DNA sequencers. Molecular Ecology Resources. 2011;11(5):759-769.

10. de Souza DJ, Bézier A, Depoix D, Drezen J-M, Lenoir A. Blochmannia endosymbionts improve colony growth and immune defence in the ant Camponotus fellah. Bmc Microbiol. 2009;9(1):29.
