# Supplementary figures and images for: Deep Sequencing Uncovers Caste-Associated Diversity of Symbionts in the Social Ant Camponotus japonicus
Source: mBio. 2020 Apr 21;11(2):e00408-20. doi: 10.1128/mBio.00408-20 (PMC7175090; doi:10.1128/mBio.00408-20)

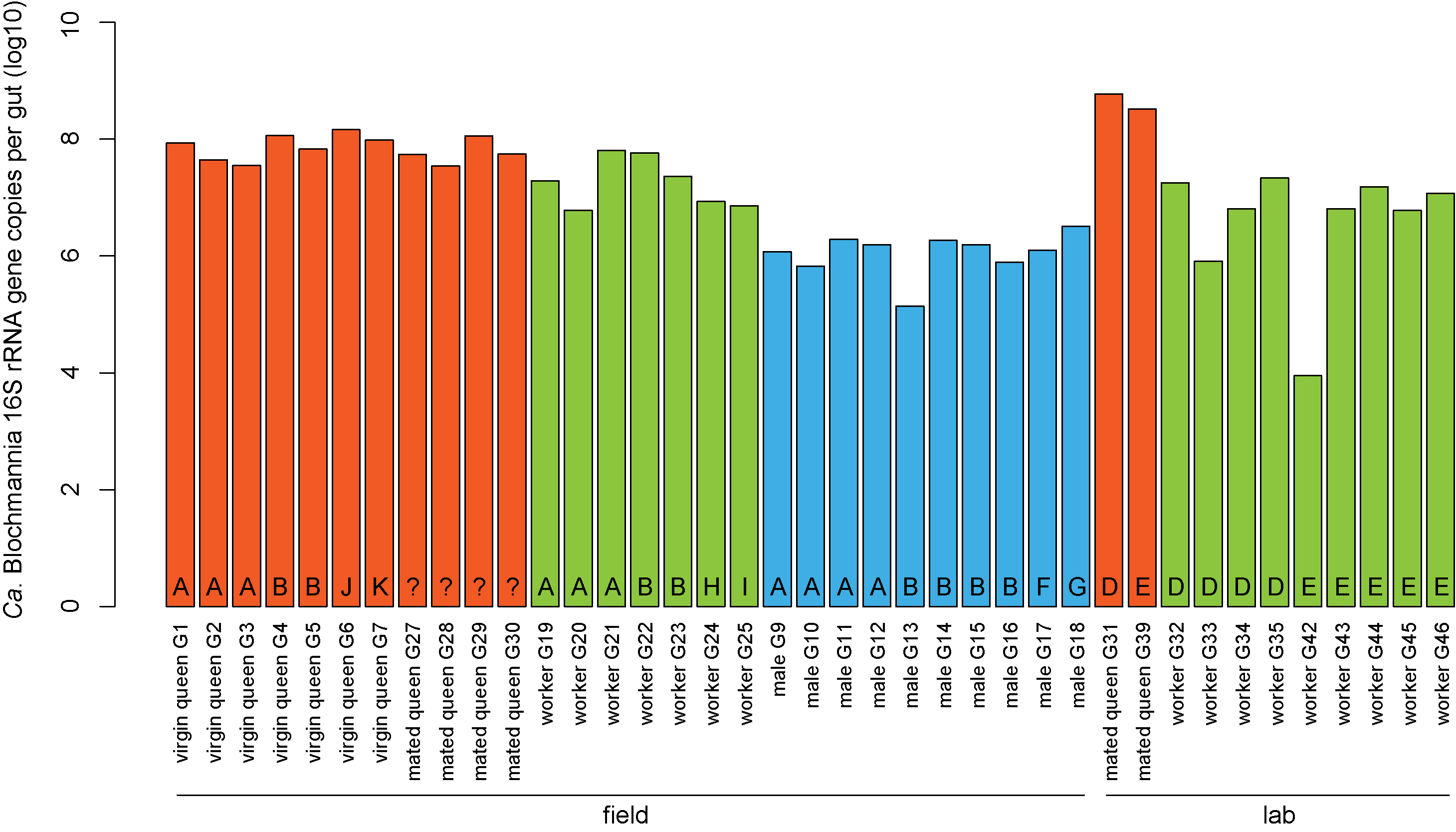

Supplement: FIG S1 [file mBio.00408-20-sf001.tif]

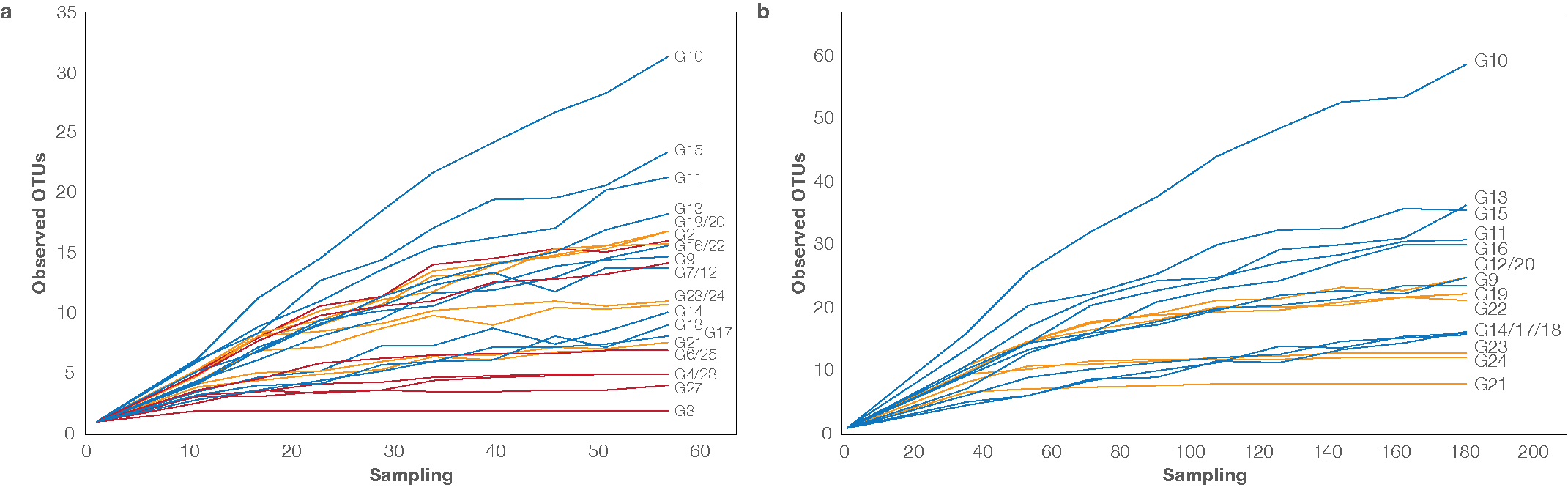

Supplement: FIG S2 [file mBio.00408-20-sf002.tif]

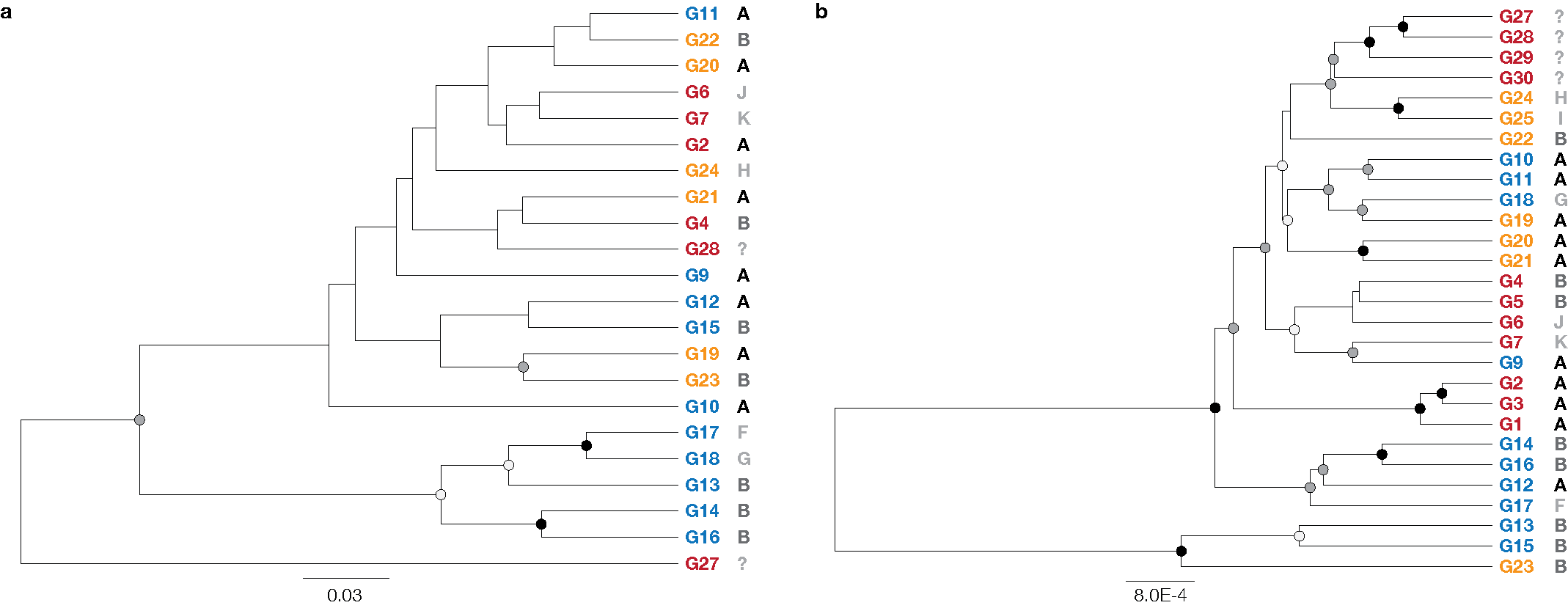

Supplement: FIG S3 [file mBio.00408-20-sf003.tif]

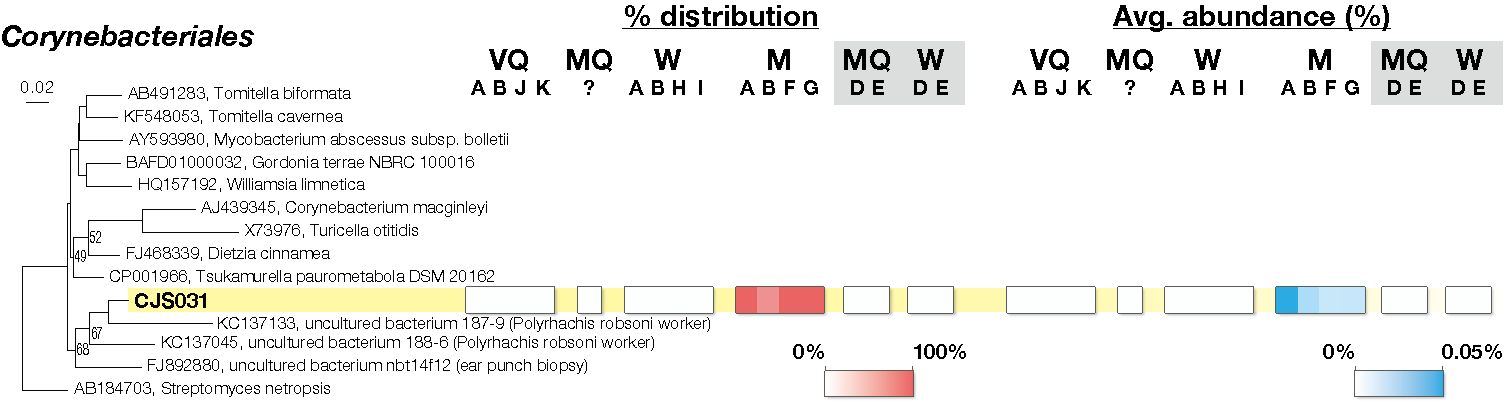

Supplement: FIG S4 [file mBio.00408-20-sf004.tif]

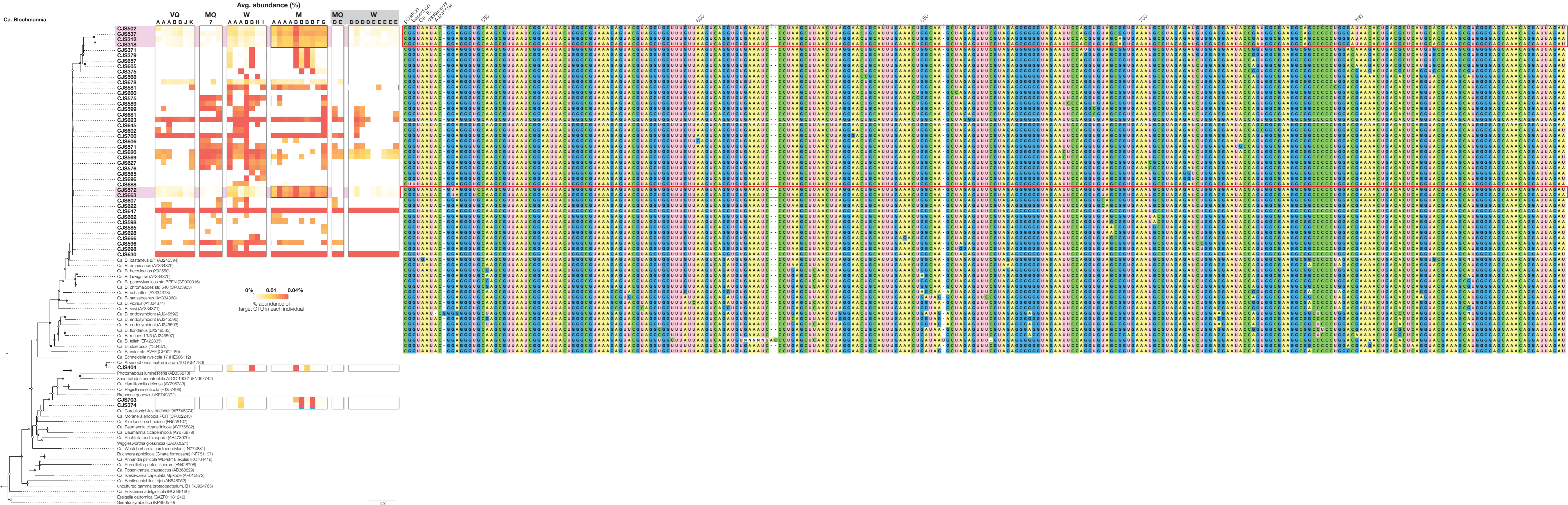

Supplement: FIG S5 [file mBio.00408-20-sf005.pdf]

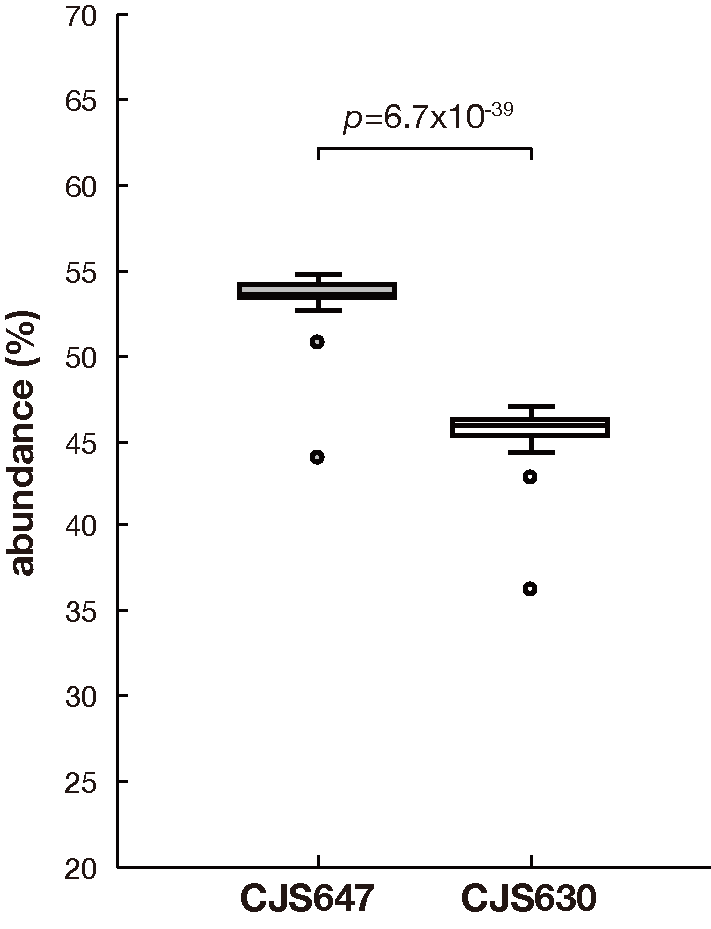

Supplement: FIG S6 [file mBio.00408-20-sf006.tif]
